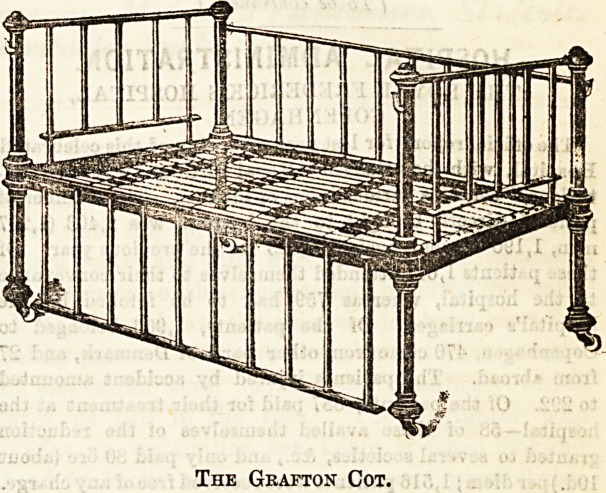# Children's Cots

**Published:** 1892-10-29

**Authors:** 


### PRACTICAL DEPARTMENTS.

### CHILDREN'S COTS.

Children's cots for hospitals or other institutions should be chosen with considerable care. It is not enough to select pretty, or strong, or cheap ones, without regard to the individual convenience of those who have to use them, and also those who have to keep them in order. No steward, contractor, or other official can judge as fairly of what a cot should be as the matron or nurse, whom practical experience has instructed in what to avoid in this matter. Therefore, in furnishing a children's hospital or a children's ward in any institution, this point ought to be taken into consideration, and the convenience of the workers and patients should be provided for. Cots must be light as well as strong, and on good castors, for the sake of the nurses and for the protection of the floor. A marked and scratched floor is plain

Atkinson's Dropsides Cots.

evidence of a faulty cot. There is no advantage in having low bedsteads for little patients, and it is needlessly wearying to their doctors and attendants. A reasonably high cot is more convenient, and generally more cheerful, too, as it gives a better chance of a window view such as children love. At the hospital for Incurable Children, in Cheyne Walk, the windows are specially designed to enable every bed-ridden child to have a view of the world outside the ward, but of course this arrangement is not by any means universal. Then as regards appearance (and this cannot be dismissed as of small importance) there is much to be said. Brass cots are charming to look at, but they need a great deal of regular "rubbing up," and they ought not to be indulged in unless the staff of workers be exceptionally large. In small wards, where there is plenty of time for the

The Grafton Cot.

ward-maid to do the polishing, or in institutions where the staff is sufficient, we have nothing to say against these pretty little bedsteads; but if nurses and probationers have "to do the brasses," we should certainly say, "Don't have them." Give up brass knobs and rails, and be content with well-shaped cots and trust to the white sheets, pretty counterpanes, and especially to the little occupants, to give the best of all adornments to the big nursery.

We need only refer to the awkward sides of the old-fashioned cots, which could not be removed except by unscrewing the knobs at each corner, and then lifting out the whole piece, which always gave a great deal of trouble to replace, and shook the framework more than was by any means desirable for a sick child. These have been succeeded by greatly improved ones. First there is the arrangement of "drop-sides," of which we give an example made by Atkinson, Westminster Bridge Road.

It is very strong, and made with either brass knobs or iron ones; it has good castors and wove-wire spring bottom—a very neat and clean arrangement, needing only a good mattress to make it a very complete bed. It is chiefly suited for children suffering from accidents or surgical diseases, as these cases being confined by splints or other restrictions are unable to unfasten the sides themselves, and therefore it is left for the nurse at bed-making time, or when the doctor is due, to let down the sides. For healthy children, or, in fact, for any little children who have the use of their hands and feet, this cot is not so safe. To unhook the sides is a diversion of a very congenial sort, and the attempts to achieve this small piece of mischief occupy many a stray moment. There are few hooks, screws, nuts, &c., which a little child's fingers cannot loosen, provided he has opportunity; and when the feat is accomplished, and the side gives way, the little urchin has every opportunity of being himself precipitated on to the floor, to the annoyance of himself and to the alarm and humiliation of his unsuspicious attendant.

Only the other day we saw in a children's ward at a country infirmary various cots which were disfigured by pieces of bandages, but certainly no visitor could take exception to this novel "decoration," after learning that it was the only method in which the safety of the small paupers could be secured in their "drop-sided" cots. If some of these poor little waifs and strays have limited mental capacity, they certainly have an unlimited capacity for mischievous deeds.

For such children, therefore, as have the free use of their limbs, the "Grafton Cot" is a safer harbour; it is also made by Messrs. Atkinson, and the side rails fall or rather slide down, and even if one end of the bar were displaced, a child could not manage to move the sides which enclose him so safely.

*(To be continued.)*

## Figures and Tables

**Figure f1:**
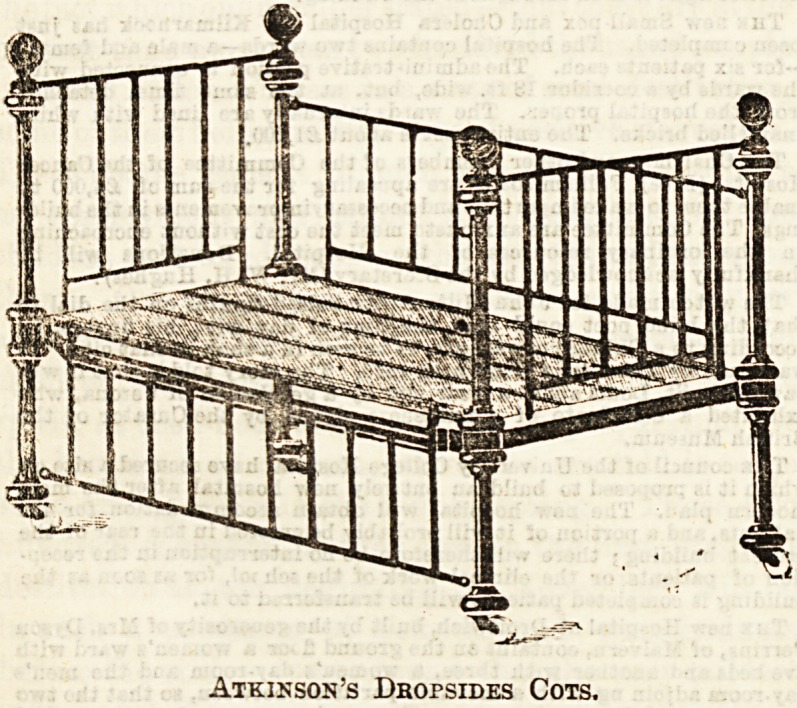


**Figure f2:**